# Nrf2 de-SUMOylation alleviates myocardial ischemia-reperfusion injury (MIRI) by attenuating myocardial ferroptosis in mice

**DOI:** 10.1080/13510002.2026.2624946

**Published:** 2026-02-06

**Authors:** Qinyun Shi, Weifeng Yao, Wenlong Zhang, Jiaqian Xu, Xiyu Wang, Xiangyun Wei, Shuming Hu, Qiuju Fan, Huan Yang, Xiaoling Wu, Rong Cai

**Affiliations:** a Department of Biochemistry & Molecular Cell Biology, Shanghai Jiao Tong University School of Medicine, Shanghai, People’s Republic of China; b Department of Cardiovascular Medicine, Shanghai Baoshan District Wusong Central Hospital, Shanghai, People’s Republic of China; c Changning Administration Center of Public Hospital and Community Healthcare Center, Shanghai, People’s Republic of China; d The Central Laboratory, Shanghai Eighth People's Hospital, Shanghai, People’s Republic of China

**Keywords:** MIRI, Ferroptosis, Nrf2, SUMOylation, SENP1, K110R, RSL_3_, Liproxstain-1

## Abstract

**Objectives:**

Reperfusion, an essential therapeutic strategy for salvaging ischemic myocardium in ischemic heart disease, paradoxically exacerbates myocardial injury. Ferroptosis is a pivotal mechanism underlying myocardial ischemia-reperfusion injury (MIRI). Nrf2 can regulate ferroptosis, which could undergo SUMOylation at lysine 110 (K110) and was subsequently de-SUMOylated by Senp1. This study aimed to determine whether Nrf2 de-SUMOylation could mitigate MIRI by inhibiting myocardial ferroptosis.

**Methods:**

Nrf2 K110R mice, mimicking Nrf2 de-SUMOylation, were generated. Mice cardiac morphology and function were observed by hematoxylin–eosin staining (HE) and echocardiography under normal and MIRI conditions. Ferroptosis inhibitor liproxstatin-1 (Lip-1) was used to demonstrate ferroptosis participation in Nrf2 de-SUMOylation regulated MIRI. *In vitro,* SUMO1/sentrin-specific protease 1 *Senp1* KO H9C2 cells were subjected to RSL_3_-induced ferroptosis to explore underlying mechanism.

**Results:**

Nrf2 K110R mice showed normal cardiac morphology and function at baseline. However, de-SUMOylation of Nrf2 alleviated myocardial ferroptosis, resulting in a reduction of MIRI severity in MIRI mice. The administration of Lip-1 attenuated the differences in MIRI between Nrf2 wild-type and K110R mice. Mechanistically, Nrf2 de-SUMOylation was associated with a reduction in Transferrin receptor (Tfr) expression level, thereby mitigating ferroptosis in cardiomyocytes.

**Conclusion:**

This study highlighted the role of Nrf2 SUMOylation in promoting ferroptosis during MIRI and identified Nrf2 de-SUMOylation as a potential therapeutic target for MIRI.

## 1. Introduction

Ischemia heart disease remains a leading cause of death and disability worldwide [1,2]. During the course of treatment, although timely coronary reperfusion can effectively salvage ischemic myocardium, the restoration of blood flow during reperfusion may paradoxically contribute to additional myocardial damage [1–3]. Cardiomyocyte death is a principal factor underlying myocardial ischemia-reperfusion injury (MIRI). To date, various forms of regulated cell death, including apoptosis, necroptosis, ferroptosis, and pyroptosis, have been identified as key contributors to MIRI [4,5]. Among them, ferroptosis has emerged as the predominant form of cell death in the later stages of MIRI [6–8].

Ferroptosis was first characterized by the laboratory of Brent R. Stockwell in 2012 [9], as a form of iron-dependent cell death driven by lipid peroxidation [10]. In recent years, accumulating evidence has elucidated the significant role of ferroptosis in the pathophysiology of MIRI [6,11–15]. Notably, inhibiting ferroptosis during MIRI has demonstrated remarkable cardioprotective effects [11,16,17], thereby highlighting the potential of targeting ferroptosis as a novel therapeutic strategy for MIRI.

The biological function of Nrf2, a critical cellular antioxidant transcription factor, in ferroptosis is well-documented [18–20]. Given its fundamental role in mitigating oxidative stress, Nrf2 has been shown to counteract ferroptosis in various disease models [21–24]. In the context of MIRI, Nrf2 typically exerts protective effects [25–28]. However, in a groundbreaking study published in 2019, it was reported that Nrf2 exacerbates doxorubicin (DOX) – and ischemia-reperfusion-induced cardiomyopathy in mice. This study, for the first time, revealed that Nrf2 could aggravate cardiomyocyte ferroptosis in MIRI through the upregulation of *Hmox-1* (*Ho-1*) expression level, thus contributing to the pathogenesis of MIRI [11].

In our previous study, it was demonstrated that the human NRF2 protein undergoes modification by SUMO1 at lysine 110, and this modification site is conserved between mice and humans [29]. SUMO1 modification enhances NRF2’s transcriptional activity by recruiting the co-activator CHD6 [29]. Furthermore, it was identified that SUMO-specific protease 1 (SENP1) is responsible for the de-SUMOylation of NRF2 [29]. SENP1 has previously been shown to protect against MIRI through a HIF-1α-dependent mechanism [30]. Recent studies have further highlighted the role of SUMOylation in cardiovascular diseases [6,31]. In the present study, Nrf2 K110R mice were generated to investigate the role of Nrf2 de-SUMOylation in the pathophysiology of MIRI. The findings elucidated that Nrf2 de-SUMOylation could alleviate MIRI by reducing cardiomyocyte ferroptosis through the downregulation of transferrin receptor (Tfr) expression level.

## 2. Materials and methods

### 2.1. Reagents and antibodies

The reagents and antibodies used in this study are described in Table 1.

**Table 1.** Antibodies and main chemicals and reagents used in this study.

Reagents and Antibodies	Identifier	Source
NRF2 (D1Z9C) XP Rabbit mAb	12721S	Cell Signaling Technology
Anti-Rabbit SENP1	ab108981	Abcam
TFRC/CD71 Rabbit Polyclonal Antibody	AF8136	Beyotime
Heme Oxygenase 1 (HO-1) Rabbit Monoclonal Antibody	AF1333	Beyotime
Anti-SUMO-1 antibody produced in a rabbit	S8070	Sigma-Aldrich
Anti-Ferritin Antibody	AF2104	Beyotime
Anti-Glutathione Peroxidase 4 antibody	ab125066	Abcam
xCT/SLC7A11 (D2M7A) Rabbit mAb	26864-1-AP	Proteintech
Beta Actin Mouse Monoclonal Antibody	60008-1-Ig	Proteintech
Enhanced BCA Protein Assay Kit	P0009	Beyotime
Lipid Peroxidation MDA Assay Kit	S0131S	Beyotime
KAPA Stranded RNA-Seq Library Prep kit	KK8400	Illumina
rProtein A/G Magarose Beads	SM005002	Smart-lifesciences
TRIzol Universal Reagent	DP424	TIANGEN
HiScript III All-in-one RT SuperMix Perfect for qPCR	R333-01	Vazyme
ChamQ Universal SYBR qPCR Master Mix	Q711-03	Vazyme
RSL3	S8155	Selleck
Liproxstatin-1	S7699	Selleck
Protease Inhibitor Cocktail	B14001	Selleck
BODIPY 665/676 (Lipid Peroxidation Sensor)	B3932	Invitrogen
Cell Counting Kit-8	CK04	DOJINDO
Iron Assay Kit -Colorimetric	I291	DOJINDO
TTC staining kit	A610558	Sangon Biotech
Hematoxylin-Eosin (HE) staining kit	E607318	Sangon Biotech
Alcian blue staining solution	TMS-010-C	Sigma
Citrate Antigen Retrieval Solution	E673001	Sangon Biotech

### 2.2. Animals

**Figure 1.**
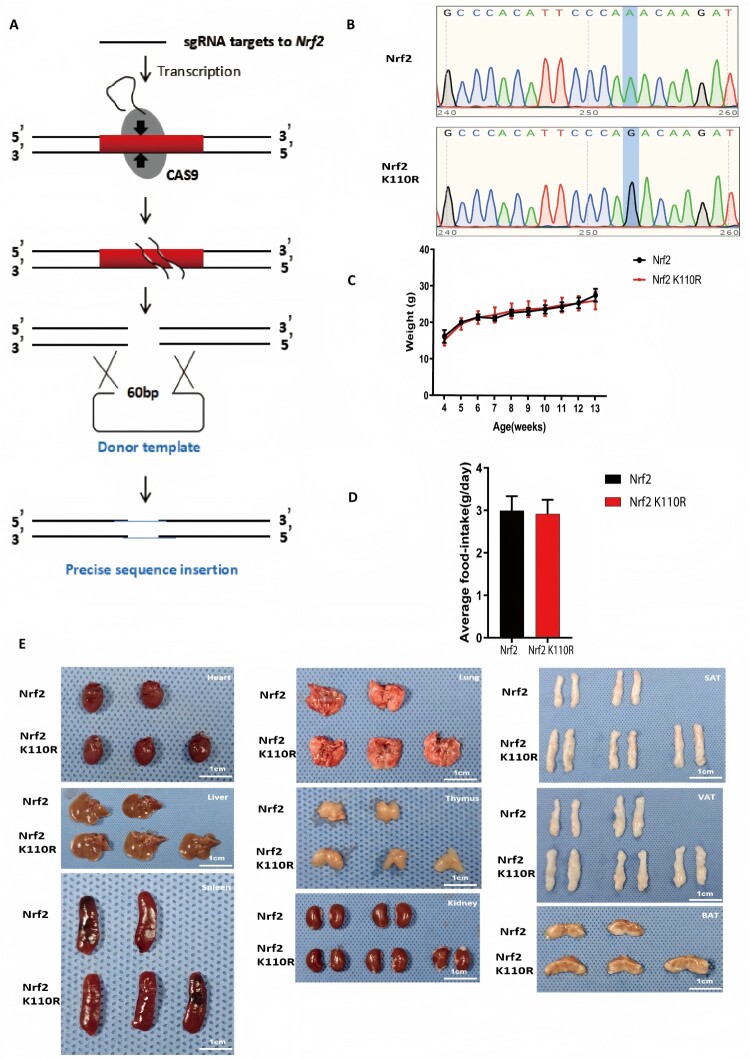
Nrf2. K110R mice displayed no disparity in comparison with Nrf2 wild-type mice under normal conditions. (A) Construction workflow of Nrf2 K110R mouse by CRISPR/Cas9. (B) The genotyping sequence of Nrf2 wild-type or Nrf2 K110R mouse. (C) Nrf2 K110R mice exhibited no difference in body weight from wild-type littermates (n = 6). (D) Nrf2 K110R mice showed no difference in food intake from wild-type littermates (n = 6). (E) Nrf2 K110R mice exhibited no difference in main organ morphology from wild-type littermates (n = 6). Data are presented as mean ± SD. Statistical significance was determined by unpaired t-test.

The animal experiments of the study were approved by the Animal care Committee of School of Medicine, Shanghai Jiao Tong University (RA-2025-078, 2025-02-24, Shanghai, China) and all experiments were performed following the Guidelines of the Care and Use of Laboratory Animals issued by the Chinese Council on Animal Research. The NFE2L2 gene (201 transcript) K110R (exon3) point mutation mice were generated utilizing CRISPR-Cas9 technology (Figure 1A). A guide RNA targeting the *NFE2L2* gene locus, ending in the NGG sequence, was designed and cleaved at the locus by CRISPR-Cas9 for *in vitro* construction. In the experimental groups, Nrf2 K110R mice were compared with wild-type littermates, and Liproxstatin-1 was administered to both groups. For clarity, the Liproxstatin-1 treatment was included as part of the groupings during the study to assess its effects on MIRI. The exogenous donor DNA was subsequently inserted into the cleavage site via homologous recombination. Only 8-week-old male mice were used in this study. Mice were randomly assigned to experimental groups using a random number table. In the MIRI model, ferroptosis was inhibited by administering Liproxstatin-1 (10 mg/kg, S7699, Selleck, USA) to mice via intraperitoneal injection once daily for a week post-surgery. Following the treatment period, animals were euthanized by exsanguination under general anaesthesia with isoflurane to obtain cardiac tissue for hypertension measurements and immunohistochemical analysis. Investigators performing surgeries, echocardiographic assessments, TTC staining, and histological analyses were blinded to group allocation. Animals that died during surgery or did not show adequate ischemia (ST-segment elevation on ECG) were excluded from the study.

### 2.3. Hematoxylin–eosin (HE) staining

The HE staining was conducted following standard procedures of tissue dewaxing and rehydration. Initially, tissue slides were incubated in hematoxylin staining solution(E607318, Sangon Biotech, China) for 1 min, followed by rinsing with running water for 5 min and immersion in 80% (v/v) alcohol for 2 min. Subsequently, the slides were stained with eosin solution(E607318, Sangon Biotech, China) for 10 sec, followed by sequential treatments with 95% (v/v) alcohol for 30 sec and 100% alcohol twice for 2 min each. The slides were thereafter subjected to two rounds of 5-min immersion in xylene. After the staining process, slides were mounted using neutral gum, air-dried for 3 h in a fume hood, and finally examined and photographed under a Leica light microscope (Leica, Germany).

### 2.4. RNA-sequencing

Total RNA samples were enriched using oligo (dT) beads, followed by library construction via the KAPA Stranded RNA-Seq Library Prep kit (KK8400, Illumina, USA). The resulting libraries were assessed for quality and size distribution using the Agilent 2100 Bioanalyzer and quantified by quantitative polymerase chain reaction (qPCR). After library construction, sequencing was carried out on an Illumina NovaSeq 6000 sequencer. Image processing and base calling were conducted using the Solexa pipeline (version 1.8). Quality control of the reads, after removal of splice junctions, was performed using FastQC software. The reads were thereafter aligned to the reference genome using Hisat2 software, and transcriptional abundance was estimated by referencing the official annotation in StringTie software. Transcriptional expression levels were analyzed using the R package Ballgown, enabling the calculation of FPKM (Fragments Per Kilobase of transcript per Million mapped reads) at both the gene and transcript levels. Differential expression was determined by comparing the genetic and transcriptional levels, with a threshold set for a 1.5-fold change, a *p*-value ≤ 0.05, and a mean FPKM in the group ≥ 0.5.

### 2.5. Mouse echocardiography

Echocardiographic assessments were performed using the Vevo 2100 high-resolution imaging system to evaluate heart function in each group of mice. Mice were first anesthetized with isoflurane (R510-22-10, RWD, China) at a concentration of 2% (v/v) in an anesthesia induction box, and then maintained anesthesia with a concentration of 1% (v/v), with the flow rate maintained at 0.4-0.6 L/min, during which both short-axis and long-axis views, along with M-mode images, were captured. At least 10 independent cardiac cycles were analyzed per experiment. Cardiac output was normalized to the animal's body weight to calculate the heart index.

### 2.6. MIRI model

Eight-week-old male mice were anesthetized by inhalation of isoflurane (R510-22-10, RWD, China), and a rodent ventilator delivering 65% (v/v) oxygen was utilized throughout the procedure. Mice were maintained at a stable body temperature of 36.5-37.5 °C using a heat lamp and heating pad, and rectal temperature was monitored regularly. Mice were divided into two groups: one group received Liproxstatin-1 (10 mg/kg, S7699, Selleck, USA) treatment, while the other did not. Namely, Nrf2 + Sham, Nrf2 K110R + Sham, Nrf2 + MIRI, Nrf2 K110R + MIRI, Nrf2 + MIRI + Lip-1, Nrf2 K110R + MIRI + Lip-1. A muscle-level incision was made between the ribs at the third intercostal space to expose the heart. Myocardial ischemia was induced by ligating the left anterior descending coronary artery using 8–0 nylon sutures. A silicone tube (1 mm OD) was placed at the base of the left coronary artery, 2 mm below the left atrium and the left ventricular (LV) border. Ischemia was confirmed by electrocardiogram (ECG) changes, specifically ST-segment elevation. Following 60-min occlusion, the silicone tube was removed to allow reperfusion.

### 2.7. Triphenyltetrazolium chloride (TTC) staining

To distinguish the ischemia area at risk zone (AAR), 1% (v/w) of Alcian blue staining solution (TMS-010-C, Sigma, USA) was perfused into the aorta and coronary arteries. Subsequently, the heart was excised, and the left ventricle (LV) was sectioned into 1 mm-thick transverse slices. These sections were incubated with 1% (v/w) TTC solution at 37 °C for 10 min. Afterward, the infarcted area (pale) and the AAR (blue) were assessed by two blinded observers. The areas were quantified using Adobe Photoshop, and the results were averaged to obtain the final measurements.

### 2.8. Malondialdehyde (MDA) measurement

The MDA content in myocardial tissue was quantified using an MDA assay kit (S0131S, Beyotime, China). Tissue samples were homogenized in RIPA lysis buffer, followed by centrifugation at 12,000 rpm (4 °C) to remove insoluble debris. A portion of the supernatant was used for BCA protein quantification (P0009, Beyotime, China), while the remaining sample was mixed with thiobarbituric acid, heated in a metal bath at 100 °C for 10 min, and centrifuged at 12,000 rpm for 10 min. The resulting supernatant was transferred to a 96-well plate, and the absorbance was measured at 532 nm using a microplate reader. The MDA content was thereafter calculated based on the standard curve, and the protein concentration was determined by the BCA assay.

### 2.9. RT-qPCR

Total RNA from cells or tissues was extracted using TRIzol Universal reagent (DP424, TIANGEN, China). RNA concentration and purity were assessed using a NanoDrop 2000c spectrophotometer. RNA was reverse transcribed into cDNA using the HiScript III All-in-one RT SuperMix Perfect for qPCR kit (R333-01,Vazyme, China) in a 20 µL reaction volume. The resulting cDNA was subsequently used for RT-qPCR analysis through the ChamQ Universal SYBR qPCR Master Mix (Q711-03, Vazyme, China). All primer sequences were validated by BLAST analysis against the mouse genome (NCBI). The specific primer sequences utilized are detailed in Table 2.

**Table 2.** Primer sequences for RT-qPCR. The primer sequences were designed according to standard criteria (e.g. target-specific, GC content, and minimal secondary structure) with product extending from 70 to 300 bp. The specificity of each primer pair was validated using the NCBI BLAST (https://blast.ncbi.nlm.nih.gov/Blast.cgi).

Target gene	Primer sequences	Accession number
18S-F	CATGCAAACCCACGACAGTA	NM_011296.3
18S-R	CCTCACGCAGCTTGTTGTCTA	
m-*Ptgs2*-F	CGGTGAAACTCTGGCTAGACAG	NM_011198.5
m-*Ptgs2*-R	GCAAACCGTAGATGCTCAGGGA	
m-*Tfr*-F	TGAGTGGCTACCTGGGCTAT	NM_011638.4
m-*Tfr*-R	CTCCTCCGTTTCAGCCAGTT	
m – *Fth1*-F	GTTGTATGCCTCCTACGTCTATC	NM_010239.2
m – *Fth1*-R	CGCTCTCCCAGTCATCACG	
m-*Gpx4*-F	GCCGTCTGAGCCGCTTACTT	NM_008162.4
m-*Gpx4*-R	GATGCACACGAAACCCCTGT	
m-*Hmox1-*F	CTAGCCTGGTGCAAGATACTG	NM_010442.2
m-*Hmox1-*R	GAAGCTGAGAGTGAGGACCC	
m-*Slc7a11*-F	ATCTCCCCCAAGGGCATACT	NM_011990.2
m-*Slc7a11*-R	GCATAGGACAGGGCTCCAAA	
Rat-*Tfr*-F	CGGCTACCTGGGCTATTGTA	XM_006248462.5
Rat-*Tfr*-R	TTCTGACTTGTCCGCCTCTT	
Rat – *Fth1*-F	GCCGAGAAACTGATGAAGCTGC	NM_012848.2
Rat – *Fth1*-R	GCACACTCCATTGCATTCAGCC	
Rat-*Gpx4*-F	GCCGAGTGTGGTTTACGAAT	NM_017165.4
Rat-*Gpx4*-R	TGGGCTGGACTTTCATCCAT	
Rat-*Hmox1*-F	TGCACATCCGTGCAGAGAAT	XM_063277779.1
Rat-*Hmox1*-R	AGGAGGCCATCACCAGCTTA	
Rat-*Slc7a11*-F	ATCTGCCCAGGACTGAGATAC	NM_001107673.3
Rat-*Slc7a11*-R	GGTGCCAATGCCCTTAGGTT	

### 2.10. Western blotting

For cell lysates, cells were thrice washed with phosphate-buffered saline (PBS) and then lysed with 200 µL of sodium dodecyl sulfate (SDS) lysis buffer containing 1% protease inhibitor for 30 min. For tissue samples, tissues were homogenized in radioimmunoprecipitation assay (RIPA) lysis buffer containing 1% (v/v) protease inhibitor (B14001, Selleck, USA). The lysate was thereafter subjected to sonication (three pulses) and heated at 95 °C for 10 min, followed by centrifugation at 12,000 rpm for 10 min. After determining the total protein concentration, 4× SDS loading buffer was added to the supernatant, and the samples were heated at 95 °C for 10 min. Equal volumes of protein samples, along with a pre-stained protein marker, were loaded onto an SDS-PAGE gel for electrophoresis.

### 2.11. Iron measurement

The colorimetric iron content detection kit (I291, DOJINDO, Japan) was used to measure ferrous ions and total iron ions in heart tissues. Firstly, fresh heart tissues were weighed, thrice washed with cold PBS, and then homogenized in 10 volumes of Assay Buffer. The samples were subjected to ultrasound treatment for 5 min. The resulting homogenate was aliquoted into three tubes (400 µL each): blank, divalent iron, and total iron sample tubes. In the total iron sample tube, 20 µL of Reducer Buffer was added, while the blank and divalent iron sample tubes received 20 µL of Assay Buffer. The samples, along with standard tubes, were incubated at 37 °C for 15 min. Subsequently, 105 µL of each sample, along with 100 µL of Probe Solution, were transferred to a 96-well plate. The plate was incubated at 37 °C for 60 min, and absorbance was measured at 593 nm using a microplate reader.

### 2.12. Cell culture

H9C2 rat cardiomyocytes were purchased from the Cell Bank of Type Culture Collection of the Chinese Academy of Sciences. All cells were incubated in a Dulbecco's modified Eagle’s medium (DMEM; D0822, Sigma-Aldrich, USA) containing 10% heat-inactivated fetal bovine serum (FBS; A5256701, Gibco, USA) and 1% penicillin/streptomycin (PS; 15140-122, Gibco, USA) at 37 °C in a humidified atmosphere containing 5% (v/v) CO_2_. The SENP1 knockout (KO) H9C2 cells were generously provided by Dr. Quan Zheng. Briefly, single-guide RNAs (sgRNAs) were designed using the CRISPick online tool (Broad Institute, https://broad.io/crispick) and subsequently cloned into the pLV [CRISPR] vector (VectorBuilder, VB230515-1168hfr). Lentivirus production was carried out by co-transfecting 293 T cells with the pLV [CRISPR] vector and the packaging plasmids psPAX2 and pMD2.G, in a 4:2:1 ratio. For effective viral infection, H9C2 cells were treated with the lentivirus in the presence of 8 mg/mL polybrene, followed by a stringent selection process using 1 mg/mL puromycin for 4–6 days. The sgRNA sequence used was TATAACCCAGACTATCACTC.

### 2.13. RSL3 treatment

H9C2 cells (NC and SENP1 KO) were treated with either DMSO or RSL_3_ (0.3 μM, S8155, Selleck, USA) for 8 h or overnight at 37 °C in a 5% CO_2_ incubator. Further analyses are detailed in the subsequent sections.

### 2.14. Immunoprecipitation

For SUMOylation immunoprecipitation assay, cells were lysed with RIPA lysate containing 1% (v/v) protease inhibitor and 20 μM N-ethylmaleimide. The protein extraction process was as previously described. After determining the protein concentration, 1 μL of the appropriate primary antibody was added to an equivalent amount of protein, and the mixture was incubated on a rotating shaker at 4 °C overnight. The following day, 20 μL of Protein A/G magnetic beads (SM005002, Smart-lifesciences, China) were added, and the mixture was incubated on a rotating shaker at 4 °C for 2 h. After magnetic separation and removal of the supernatant, the beads were thrice washed with RIPA buffer. Finally, 2 × SDS loading buffer was added, and the samples were heated in a metal bath at 95 °C for 20 min. The samples were thereafter analyzed by Western blotting.

### 2.15. Cell-counting-kit-8 (CCK8) test

Cell viability was assessed using the CCK-8 assay. A total of 20,000 cells were seeded per well into a 96-well plate, with three replicate wells per condition, each containing 100 μL of cell suspension. After overnight incubation at 37 °C, cells were treated with either DMSO or RSL3 for 8 h. Following treatment, 10 μL of CCK-8 reagent (CK04, DOJINDO, Japan) was added to each well, and the plate was incubated at 37 °C for an additional 2 h. Absorbance was measured at 450 nm using a microplate reader. Relative cell viability for each group was calculated based on the absorbance values.

### 2.16. Lipid hydroperoxide (LPO) test

H9C2 cells in 6-well plates were treated overnight with DMSO or RSL3. The following day, cells were twice washed with 1 × PBS. The cells were thereafter incubated with 5 µM of the BODIPY™665/676 probe (B3932, Invitrogen, USA) at 37 °C, 5% (v/v) CO₂ for 30 min. Afterward, the cells were collected, centrifuged at 800 rpm for 5 min, and twice washed with 1x PBS. The cells were resuspended in FACS buffer, and lipid peroxidation was quantified by flow cytometry (excitation: 665 nm, emission: 676 nm).

### 2.17. Statistical analysis

*2.5* Data were analyzed and plotted using GraphPad Prism 10, Adobe Photoshop, and Image J software. Experimental results were presented as the mean ± standard deviation (SD) from at least three independent replicates. The sample size (n) for each experiment is indicated in the figure legends. Normality of data distribution was assessed using the Shapiro–Wilk test, and homogeneity of variances was evaluated using Levene's test. For comparisons between two groups, unpaired Student's t-test was used for normally distributed data; otherwise, the Mann–Whitney U test was applied. For multiple group comparisons, one-way or two-way ANOVA followed by Tukey's post-hoc test was used for data with normal distribution and equal variances. For experiments involving inhibitor treatments, the respective control and treatment groups (e.g. Liproxstatin-1 treated vs. non-treated) were compared. Statistical significance was determined by comparing groups using appropriate tests, and a *p*-value < 0.05 was considered statistically significant.

## 3. Results

### 3.1. Nrf2 k110r mice displayed no disparity in comparison with nrf2 wild-type mice under normal conditions

To explore the role of Nrf2 de-SUMOylation *in vivo*, a Nrf2 K110R mouse model, corresponding to the human NRF2 K110R mutation, was generated using CRISPR/Cas9 technology (Figures 1A and 1B). Under normal conditions, no significant differences were identified in body weight or food intake between Nrf2 K110R mice and their wild-type littermates (Figures 1C and 1D). Additionally, there were no significant differences in the morphology or weight of major organs (Figure 1E), including the heart, liver, spleen, thymus, kidneys, lungs, and both white and brown adipose tissues, between Nrf2 K110R mice and wild-type controls (Supplementary Figure S1A). Histological examination revealed no discrepancies in organ morphology between Nrf2 K110R mice and their wild-type littermates (Supplementary Figure S1B).

### 3.2. Cardiac morphology and function of nrf2 k110r mice exhibited no disparity compared with nrf2 wild-type mice

**Figure 2.**
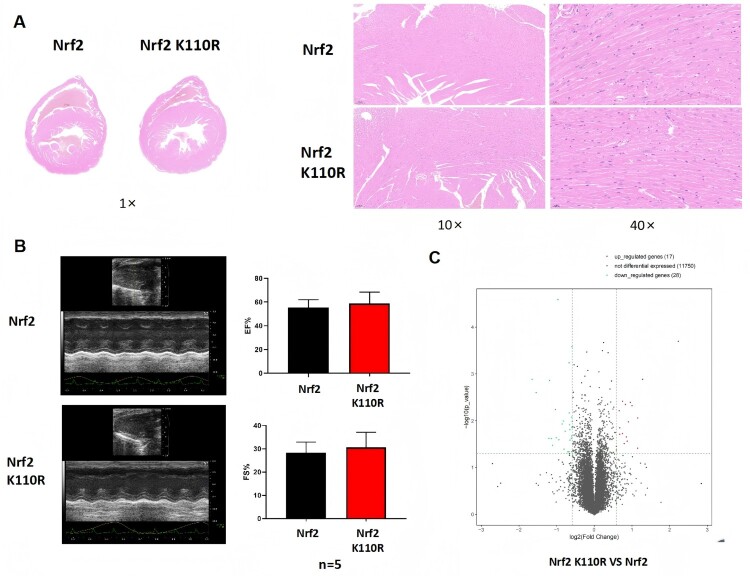
Cardiac histological morphology and function of Nrf2 K110R mice exhibited no disparity in comparison with Nrf2 wild-type mice. (A) Nrf2 K110R mice showed no difference in cardiac histological morphology from wild-type littermates. (B) Nrf2 K110R mice showed no difference in cardiac function from wild-type littermates(n = 5). (C) Nrf2 K110R mice exhibited no significant difference in myocardium transcriptome sequencing from wild-type littermates. Data are presented as mean ± SD. Statistical significance was determined by unpaired t-test.

Histological analysis using HE staining and echocardiography revealed no differences in cardiac morphology or function between Nrf2 K110R mice and their wild-type littermates (Figures 2A and 2B). Furthermore, RNA sequencing data demonstrated no significant alterations in the gene expression profile of the hearts from Nrf2 K110R mice compared with wild-type controls (Figure 2C). These findings collectively demonstrate that the loss of SUMO1 modification at lysine 110 did not affect cardiac morphology or function under normal conditions.

### 3.3. Nrf2 de-SUMOylation could protect mice against MIRI

**Figure 3.**
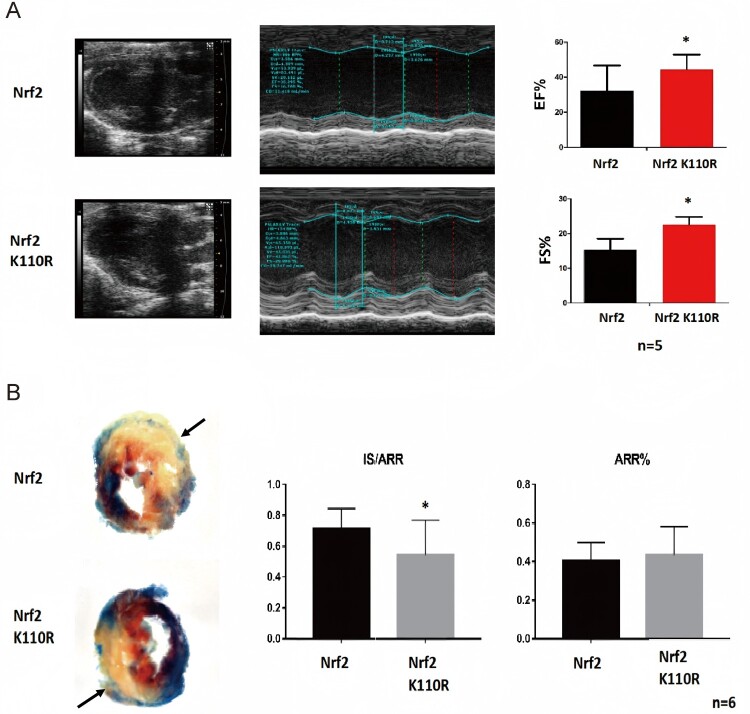
Nrf2. de-SUMOylation could be protective for mice against myocardial ischemia-reperfusion injury (MIRI). (A) Nrf2 K110R mice displayed better cardiac function than wild-type littermates when subjected to I/R (n = 5). (B) Nrf2 K110R mice exhibited less infarct size than wild-type mice when subjected to I/R (n = 6). Data are presented as mean ± SD. *p < 0.05, **p < 0.01 by unpaired t-test.

In order to elucidate the role of Nrf2 de-SUMOylation in MIRI in mice, 8-week-old male mice were subjected to ischemia operation by ligating the anterior descending coronary artery. After 1-h occlusion, the silicone tube was removed for reperfusion. Following 24-h reperfusion, echocardiography was conducted to examine the LV function of mice. Notably, Nrf2 K110R mice displayed superior ejection fraction (EF%) and LV fractional shortening (FS%) to wild-type mice (Figure 3A). Additionally, Evans blue and TTC staining revealed that Nrf2 K110R mice had a smaller infarct size in the area at risk (AAR) compared with wild-type mice, as indicated by the arrow (Figure 3B). These results confirmed that the loss of Nrf2 SUMO1 modification could mitigate MIRI in mice.

### 3.4. Nrf2 de-SUMOylation could alleviate MIRI by reducing myocardial ferroptosis

**Figure 4.**
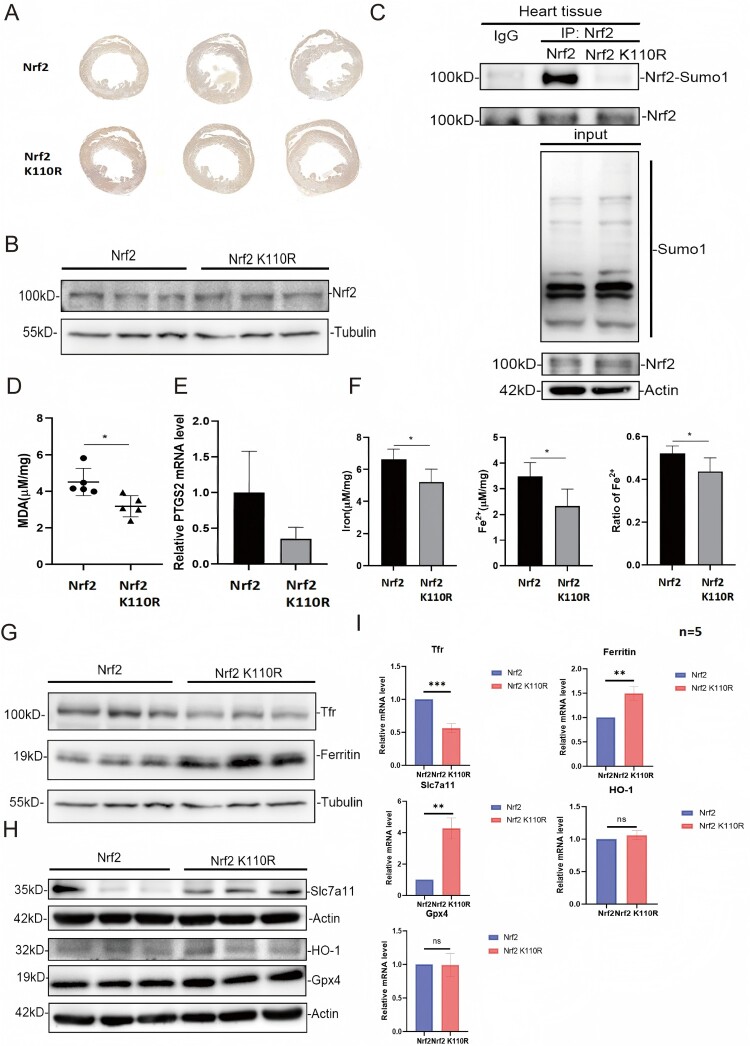
Nrf2 de-SUMOylation could alleviate myocardial ischemia-reperfusion injury by attenuating myocardial ferroptosis. (A) Nrf2 K110R mice showed no significant difference in cardiac Nrf2 expression level from wild-type mice under normal conditions, as shown by immunohistochemistry (n = 5). α-Tubulin was used as loading control. (B) Nrf2 K110R mice exhibited no significant difference in cardiac Nrf2 expression level from wild-type mice under normal conditions indicated by the arrow, as evidenced by Western blotting. (C) Nrf2 K110R mice heart tissue had lower Nrf2 SUMOylation level than wild-type mice. (D) Nrf2 K110R mice could produce lower MDA level in the myocardium when subjected to I/R (n = 6). (E) Nrf2 K110R mice had lower cardiac Ptgs2 expression level than wild-type mice when subjected to I/R (n = 5). (F) Nrf2 K110R mice exhibited lower levels of cardiac total iron ions and ferrous ions than wild-type mice (n = 6). (G) Nrf2 K110R mice have lower cardiac Tfr and higher cardiac ferritin expression level than wild-type mice when subjected to I/R. (H) Nrf2 K110R mice have higher Slc7a11 than wild-type mice. Gpx4 and Ho-1 expression exhibit no disparity between Nrf2 K110R mice and wild-type mice. Data are presented as mean ± SD. *p < 0.05, **p < 0.01 by unpaired t-test or one-way ANOVA with Tukey's post-hoc test. Ptgs2, prostaglandin-endoperoxide synthase 2; Tfr, transferrin receptor.

Although de-SUMOylation of Nrf2 did not affect Nrf2 expression during MIRI (Figures 4A, B), a notable decrease in SUMOylation level of Nrf2 and a significant reduction in MDA, a marker of lipid peroxidation, was identified in the hearts of Nrf2 K110R mice compared with wild-type mice during MIRI (Figure 4C and 4D). Similarly, the cardiac mRNA expression level of Prostaglandin-endoperoxide synthase 2 (*Ptgs2*), a marker of ferroptosis, exhibited a downward trend in Nrf2 K110R mice after MIRI (Figure 4F). Subsequently, the levels of total iron, Fe^2+^, and Fe^3+^ in the myocardial tissues of Nrf2 wild-type and K110R mice following injury were assessed. The results indicated that both total iron and Fe^2+^ levels were significantly lower in the hearts of Nrf2 K110R mice compared with wild-type mice (Figure 4E). These findings indicated a reduced severity of ferroptosis in the MIRI tissues of Nrf2 K110R mice, likely due to the absence of Nrf2 SUMO1 modification. In agreement with these findings, Western blotting and RT-qPCR analyses demonstrated reduced mRNA and protein expression level of *Tfr* and increased mRNA and protein expression level of *ferritin* and *Slc7a11* in the cardiac tissues of Nrf2 K110R mice compared with wild-type mice during MIRI, while the expression of *Ho-1* and *Gpx4* remain stable (Figure 4G–I), reflecting reduced iron uptake and enhanced iron storage in the hearts of Nrf2 K110R mice. Collectively, these results demonstrated that Nrf2 de-SUMOylation could be protective against MIRI by reducing myocardial ferroptosis.

### 3.5. Ferroptosis inhibition with liproxstatin-1 mitigated MIRI in mice induced by nrf2 SUMOylation

**Figure 5.**
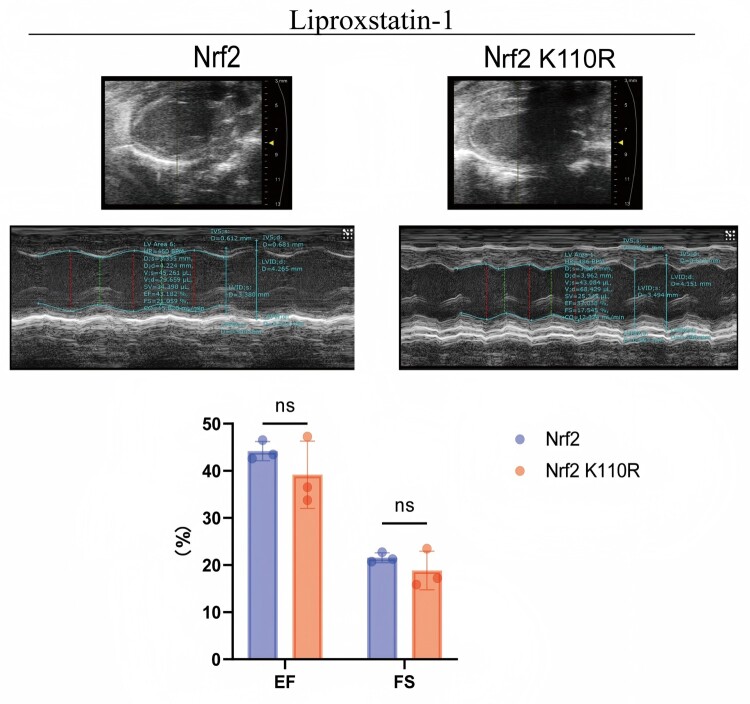
Ferroptosis inhibitor Liproxstatin-1 relieves mice myocardial ischemia-reperfusion injury promoted by Nrf2 SUMOylation. (A) A schematic timeline of MIRI model surgery and Liproxstatin-1 injection. (B) Liproxstatin-1 treatment eliminated the functional differences between Nrf2 wild-type and K110R mice during MIRI (n = 3). Data are presented as mean ± SD. *p < 0.05, **p < 0.01 by two-way ANOVA with Tukey's post-hoc test.

To further investigate the role of Nrf2 SUMOylation in inducing MIRI via ferroptosis, the ferroptosis inhibitor Liproxstatin-1 was administered to Nrf2 wild-type and K110R mice undergoing MIRI surgery. Mice were injected Liproxstatin-1 a day after surgery, extending to once a day for seven times in total, as indicated by the schematic timeline (Figure 5A). Echocardiography results revealed that treatment with Liproxstatin-1 for one week eliminated the functional differences between Nrf2 wild-type and K110R mice during MIRI (Figure 5B). This reflects that Nrf2 SUMOylation could exacerbate MIRI by enhancing myocardial ferroptosis.

### 3.6. Nrf2 SUMOylation exacerbated H9C2 cell ferroptosis induced by RSL3 by upregulating tfr expression level

**Figure 6.**
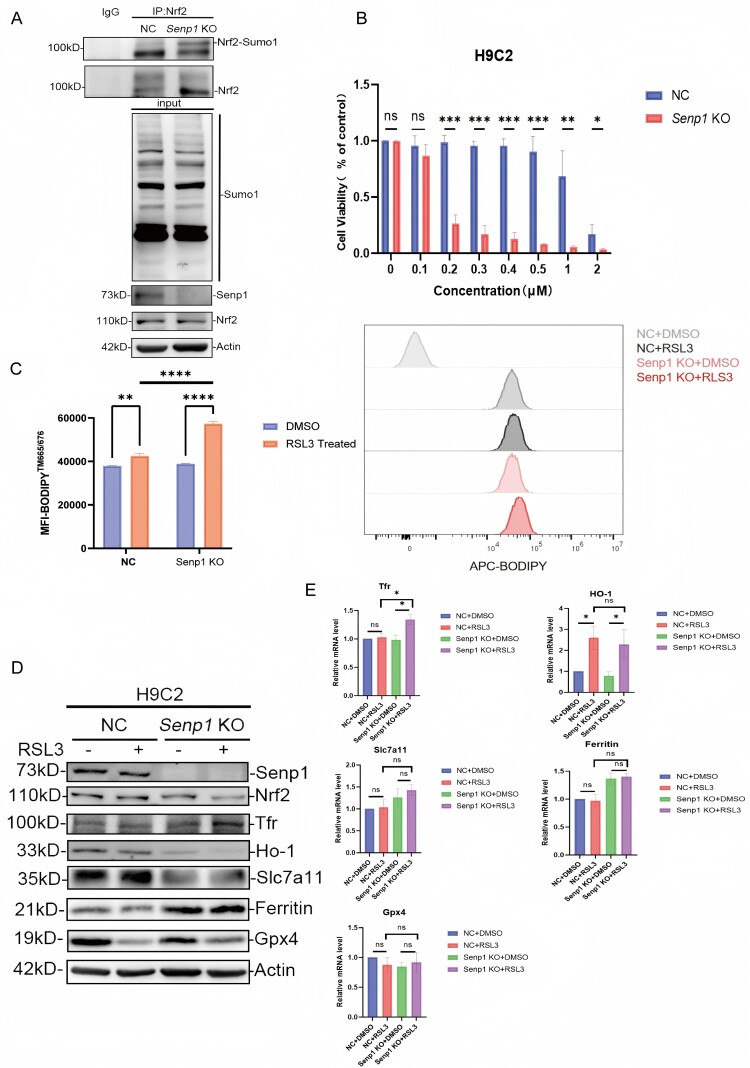
Nrf2. SUMOylation exacerbated H9C2 cell ferroptosis induced by RSL_3_ by promoting Tfr expression level. (A) Senp1 deficiency resulted in accumulated Nrf2 SUMOylation in H9C2 cells. (B) *Senp1* deficiency could cause more H9C2 cell death when treated with RSL_3_ (n = 3). (C) *Senp1* KO H9C2 cells had higher LPO than NC cells when treated with RSL3 (n = 3). (D) *Senp1* KO H9C2 cells exhibited a higher expression level of Tfr than NC cells when treated with RSL3. β-actin was used as loading control. Data are presented as mean ± SD. *p < 0.05, **p < 0.01, ***p < 0.001, ****p < 0.0001 by unpaired t-test or one-way ANOVA with Tukey's post-hoc test.

To characterize the underlying mechanism by which Nrf2 SUMOylation exacerbates ferroptosis in cardiac cells, experiments were conducted using Senp1 wild-type and knockout H9C2 cells. As previously described, the Senp1 knockout resulted in a significant accumulation of SUMOylated Nrf2 (Figure 6A). These cells were thereafter treated with the ferroptosis inducer RSL3 in a dose-dependent manner. The results demonstrated that Senp1 deficiency significantly sensitized H9C2 cells to RSL3-induced cell death (Figure 6B). Additionally, Senp1 knockout led to the increased lipid peroxidation (LPO) level in H9C2 cells during RSL3-induced ferroptosis (Figure 6C). Mechanistically, Senp1 knockout was associated with the elevated expression level of transferrin receptor (*Tfr*) in H9C2 cells following RSL3 treatment (Figure 6D), which is similar to that in the mRNA level (Figure 6E), mirroring the *in vivo* data found in Nrf2 K110R mice (Figure 4G). But Other Nrf2-downstream protein exhibit no significant difference during this RSL3-induced process. Taken together, these findings demonstrated that Nrf2 SUMOylation could exacerbate ferroptosis in cardiac cells induced by RSL3 through upregulation of *Tfr* expression level.

## 4. Discussion

On the basis of prior investigations [29], Nrf2 K110R mice were generated (Figures 1 and 2) to explore the role of Nrf2 de-SUMOylation in MIRI. The results demonstrated that Nrf2 de-SUMOylation, promoted by Senp1, could mitigate MIRI by attenuating cardiomyocyte ferroptosis (Figures 3 and 4). Notably, *in vivo* administration of the ferroptosis inhibitor Liproxstatin-1 attenuated the differences in MIRI between Nrf2 wild-type and K110R mice (Figure 5), highlighting the critical role of ferroptosis in the exacerbation of MIRI driven by Nrf2 SUMOylation.

While Nrf2 activity is known to be modulated by various post-translational modifications – including phosphorylation, acetylation, and ubiquitination – their roles in MIRI-associated ferroptosis remain context-dependent and incompletely understood. Previous studies have highlighted the importance of Keap1-mediated ubiquitination in regulating Nrf2 stability and its antioxidative function during ischemia-reperfusion [14, 15]. In contrast, our work identifies SUMOylation at the conserved K110 residue as a specific and previously underappreciated regulatory layer that directly links Nrf2 to iron metabolism and ferroptosis in cardiomyocytes. Unlike global Nrf2 activation or suppression, which may simultaneously influence numerous antioxidant and detoxification pathways, de-SUMOylation at K110 appears to preferentially modulate a subset of Nrf2 target genes involved in iron handling – most notably *Tfr*. This selectivity may explain why we observed significant changes in Tfr and ferritin without concomitant alteration in Ho-1 expression, a pattern distinct from that reported in models of doxorubicin-induced cardiomyopathy where Ho-1 plays a central role [11]. Thus, K110 de-SUMOylation functions as a molecular switch that fine-tunes Nrf2’s transcriptional output toward iron homeostasis without broadly affecting its classical antioxidant targets, offering a more precise means to intervene in ferroptosis-driven injury without disrupting redox balance [32].

*Ho-1* is a well-known target gene of Nrf2, which promotes the hydrolysis of heme and increases the concentrations of free iron ions [33]. Activation of the Nrf2/Ho-1 pathway has been implicated in iron overload and subsequent ferroptosis in cardiomyocytes, particularly in the context of doxorubicin-induced cardiomyopathy [11]. However, in the present study, no significant differences in *Ho-1* expression level were detected between the hearts of Nrf2 wild-type and Nrf2 K110R mice (data were not shown), despite its confirmed role in doxorubicin-induced myocardial ferroptosis [11]. *Ferritin*, another Nrf2-regulated gene, encodes an iron storage protein and can modulate ferroptosis and cardiomyopathy. Loss of *ferritin* has been shown to exacerbate ferroptosis and cardiac dysfunction [34–36]. In this study, a higher *ferritin* expression level was identified in the hearts of Nrf2 K110R mice during MIRI (Figure 4F), reflecting enhanced iron storage capacity in these hearts. This likely reduces the availability of free iron in cardiomyocytes, thereby mitigating ferroptosis. Additionally, reduced Tfr expression level was found in the hearts of Nrf2 K110R mice, indicating diminished iron uptake in these cells. Tfr, which has been identified as a notable ferroptosis marker [37], promotes the import of iron into cells, thereby enhancing ferroptosis by increasing intracellular iron levels [38]. Collectively, these data support the outcome that Nrf2 de-SUMOylation could alleviate MIRI by reducing myocardial ferroptosis. However, the precise molecular mechanisms by which Nrf2 SUMOylation regulates *Tfr* expression level remain to be fully elucidated. Bioinformatic analysis reveals potential ARE like motifs in the *Tfr* promoter (Supplementary Figure S2), suggesting that *Tfr* may be a direct transcriptional target of Nrf2. This possibility is strongly supported by a recent study showing that Nrf2 directly binds to an ARE within the *Tfr* promoter (site: 1516–1506 bp) in microglia under cerebral ischemia reperfusion injury, thereby transcriptionally upregulating TFR expression [39]. This parallel finding highlight a conserved role for Nrf2 in the transcriptional control of iron uptake genes across different ischemic contexts.

Previous research has demonstrated that Senp1 could be protective against MIRI through a HIF1α-dependent pathway [30]. Recent research revealed that hypoxia could induce ferroptosis in trophoblast cells, leading to miscarriage, by upregulating HIF1α-SUMO-mediated transcription of NCOA4 [40]. Additionally, SENP2, a member of the SUMO-specific protease family, has been found to protect against ferritinophagy-dependent ferroptosis in MIRI by de-SUMOylating NCOA4 [41]. Collectively, these findings highlight the notable function of SUMOylation and de-SUMOylation in the regulation of MIRI [30, 40,41]. In the current study, using Senp1-deficient H9C2 cardiomyocytes with accumulated Nrf2 SUMOylation, it was revealed that loss of *Senp1* sensitized H9C2 cells to RSL3-induced ferroptosis *in vitro* (Figure 6). Mechanistically, *Senp1* loss led to a significant elevation in Tfr expression level in H9C2 cells upon RSL3 treatment (Figure 6), which may be transcriptionally induced by accumulated Nrf2 SUMOylation. These *in vitro* findings are consistent with observations in mouse heart tissues; however, the precise mechanisms by which *Senp1* loss modulates Tfr expression level during RSL3-induced ferroptosis require further investigation. In addition, in H9C2 cells, we did not observe the disparity of *ferritin* expression during ferroptosis as in mouse heart tissues. Since the use of rat-origin H9C2 cells as a model for cardiomyocytes may not fully recapitulate the complexities of *in vivo* murine MIRI model because of their species difference consideration of alternative *in vitro* models using primary Nrf2 wild-type and K110R cardiomyocytes would be helpful.

Furthermore, our study prompts consideration of the broader regulatory landscape of Nrf2 in ferroptosis. Nrf2 is known to transcriptionally regulate multiple ferroptosis-related genes, including *Gpx4*, *Slc7a11* (encoding xCT), and Fth1 (encoding ferritin heavy chain) [19,22,23]. The expression changes of several such genes observed in our model (Fig. 6D) suggest that Nrf2 SUMOylation may exert pleiotropic effects. However, the pronounced sensitivity of Trf expression to Nrf2 SUMOylation status indicates a selective regulatory mechanism. This selectivity may arise from gene-specific promoter architectures, varying affinities for SUMO-modified Nrf2, or the involvement of distinct co-regulators at different target loci [45]. It suggests that Nrf2 SUMOylation does not uniformly amplify all its transcriptional programs but may fine-tune specific subsets of genes – particularly those governing iron uptake (Trf) – thereby precisely steering cellular fate under ischemic stress. Future genome-wide studies comparing the binding profiles of SUMOylated versus unmodified Nrf2 will be essential to map its full regulon and elucidate the determinants of this selectivity.

Our study has some limitations. First, we used only male mice, which may limit the generalizability of our findings to females. Future studies should include both sexes. Second, the H9C2 cell line is of rat origin, while our in vivo model is murine, which may account for some discrepancies. Primary cardiomyocytes or human cardiomyocyte cell lines may provide more physiological relevance. Third, we focused on *Tfr*, but Nrf2 SUMOylation may regulate other ferroptosis-related genes (FEGs), thus a more comprehensive analysis would be valuable. Finally, the long-term effects of Nrf2 de-SUMOylation on cardiac remodeling after MIRI warrant further investigation.

While previous studies have concentrated on the general role of Nrf2 in MIRI and ferroptosis, this study uniquely identified the critical role of Nrf2 de-SUMOylation in mitigating MIRI through a mechanism involving reduced myocardial ferroptosis. In contrast to earlier research that primarily linked Nrf2 activation to antioxidative defense, our findings demonstrated that the loss of Nrf2 SUMOylation exhibited a protective effect, highlighting a more detailed role of Nrf2 post-translational modification in heart disease. Moreover, by indicating that de-SUMOylation could reduce the severity of MIRI through ferroptosis modulation, a potential therapeutic strategy was proposed targeting the Nrf2 SUMOylation pathway. This outcome diverges from the traditional view of Nrf2 as simply an antioxidant regulator, instead positioning it as a key player in iron homeostasis and ferroptosis regulation, which may open new directions for clinical intervention. In terms of clinical implications, the present study demonstrated that strategies aimed at enhancing Nrf2 de-SUMOylation or inhibiting Nrf2 SUMOylation could provide novel therapeutic options for conditions, such as MIRI, where ferroptosis plays a significant role in exacerbating cardiac damage. Furthermore, the reduction in myocardial ferroptosis by targeting SUMOylation pathways may lead to the development of more effective treatments for ischemic heart diseases, where managing iron overload and oxidative stress is crucial. Therapeutically targeting Nrf2 SUMOylation presents both opportunities and challenges. Small-molecule inhibitors of the SUMOylation cascade (e.g. targeting SAE, UBC9, or specific E3 ligases) could globally reduce Nrf2 SUMOylation, but may have off-target effects due to the broad roles of SUMOylation in cellular signaling [42]. More selective approaches – such as enhancing SENP1 activity or disrupting the Nrf2-SUMO E3 interaction – could offer greater precision. Gene-therapy strategies using AAV-mediated delivery of SENP1 or SUMO-resistant Nrf2 (e.g. K110R) to the heart also hold promise for sustained, tissue-specific modulation [43]. Key challenges include achieving cardiac-specificity, minimizing immune responses, and balancing Nrf2 activity – as both excessive activation and suppression can be detrimental [44]. Future work should evaluate whether combining SUMOylation-targeted agents with classical Nrf2 activators yields synergistic cardio protection with improved safety profiles.

In conclusion, while Nrf2 SUMO1 modification could enhance Nrf2 transcriptional activity and antioxidant defense, it could exacerbate MIRI by promoting cardiomyocyte ferroptosis through upregulation of *Tfr* expression level.

## Supplementary Material

Supplementary File.docx

Checklist.pdf

## Data Availability

All data from this study are available upon reasonable request by contacting the corresponding author.
