# Supplementary material for: Nrf2 de-SUMOylation alleviates myocardial ischemia-reperfusion injury (MIRI) by attenuating myocardial ferroptosis in mice
Source: Redox Rep. 2026 Feb 6;31(1):2624946. doi: 10.1080/13510002.2026.2624946 (PMC12885000; doi:10.1080/13510002.2026.2624946)
Supplement: Supplementary File.docx [file YRER_A_2624946_SM9430.docx]

**Fig.S1.** **Nrf2 K110R mice have no differences in main organ weight and organ histological morphology.** (A) Nrf2 K110R mice have no difference in organ weight from littermates wild-type mice (n=3). (B) Nrf2 K110R mice have no difference in organ histological morphology from littermates wild-type mice.

**Fig.S2.** **Nrf2 binding sites in the *Tfr* promoter region.** (A) Binding Motif of Nrf2 (B) Schematic representation of the predicted Nrf2 binding motifs within the *Tfr* promoter. Two potential Nrf2 binding sites (site 1 and site 2) are indicated, with their relative positions to the transcription start site (+1) and predicted binding scores shown.


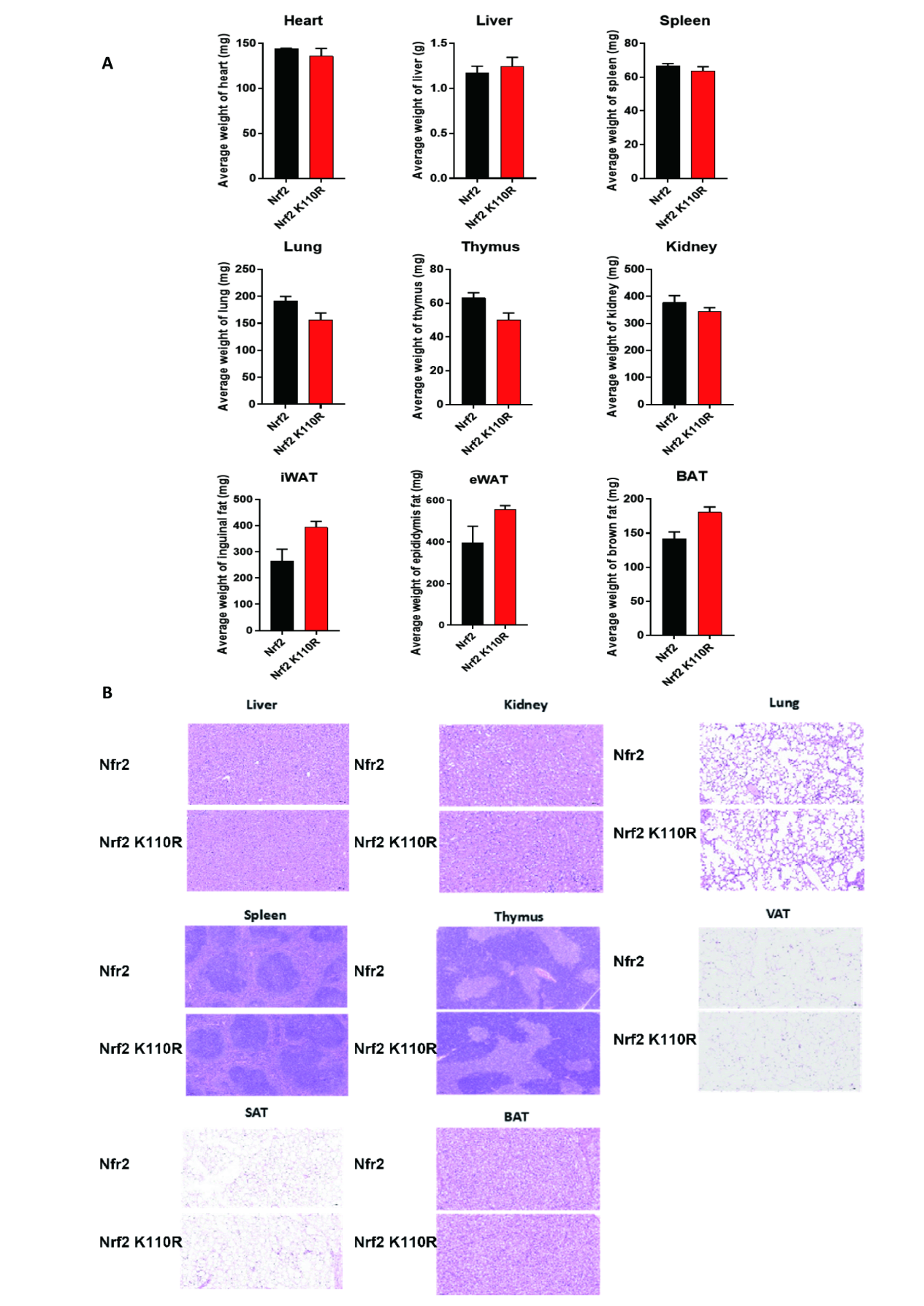


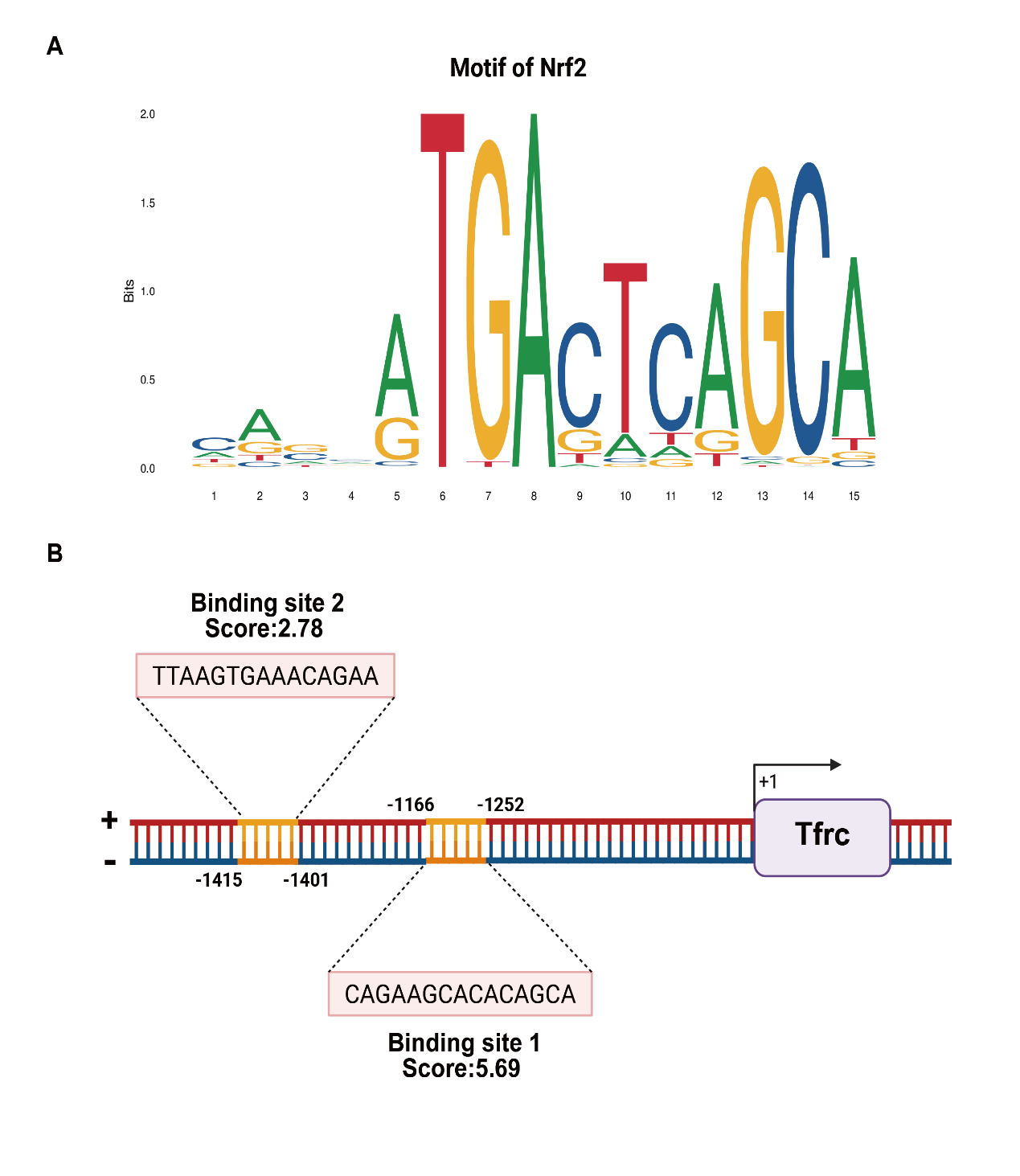


**Fig 3B 3F**


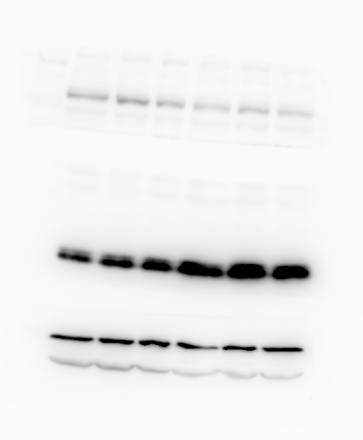

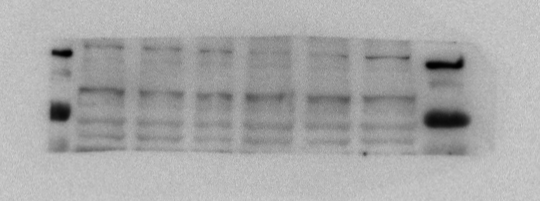


Tfr（100kD）

Ferritin（19kD）

Tubulin（55kD）

Nrf2（110kD）

**Fig3C**


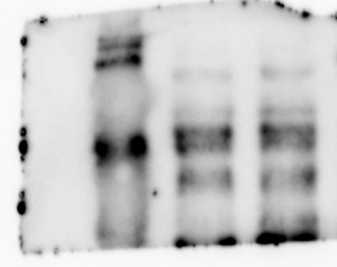

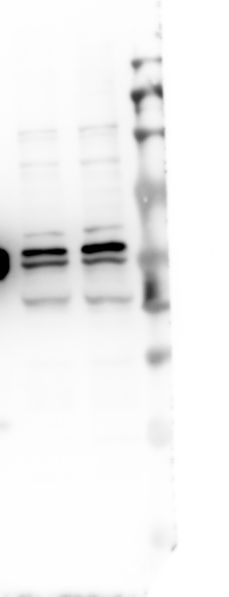

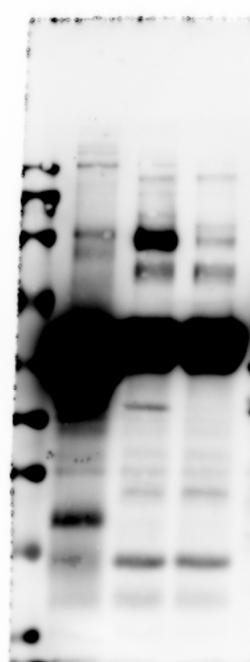

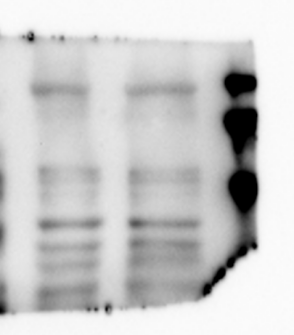

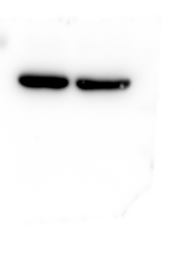


Actin（42kD）

Nrf2（110kD）

Sumo1

Nrf2（110kD）

Nrf2-Sumo1（110kD）

**Fig3H**


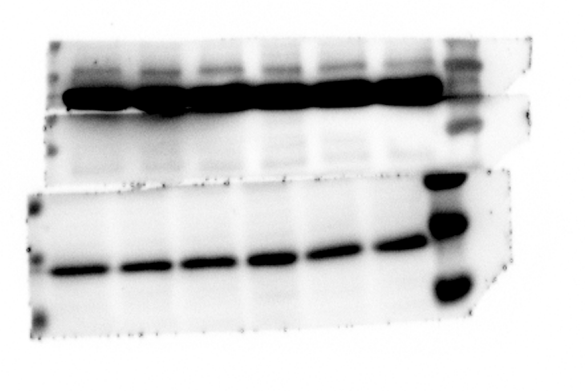

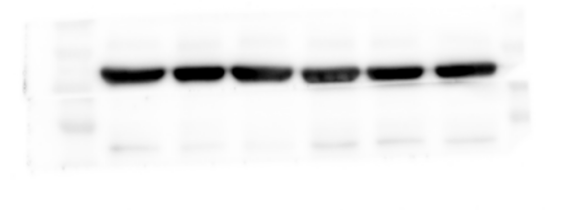


Ho-1（32kD）

Actin（42kD）

Gpx4（19kD）

Actin（42kD）

Slc7a11（35kD）

**Fig6A**


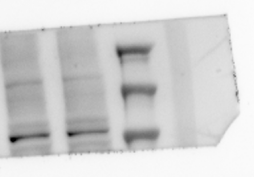

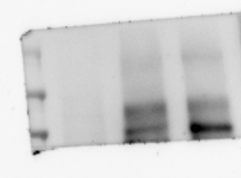

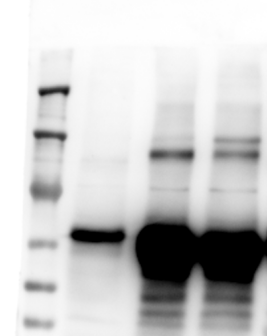

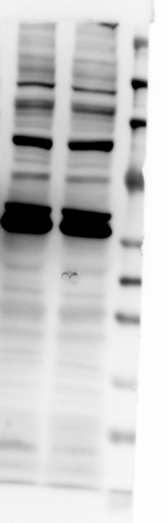

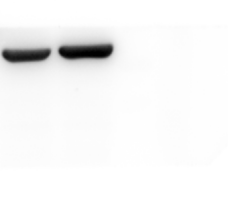

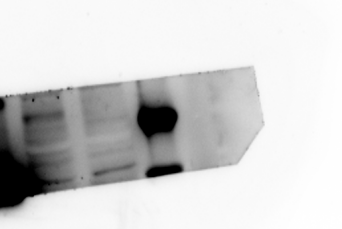


Nrf2-Sumo1（110kD）

Nrf2（110kD）

Nrf2（110kD）

Senp1（73kD）

Actin（42kD）

SUMO1

**Fig6D**


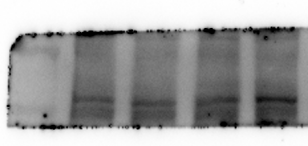

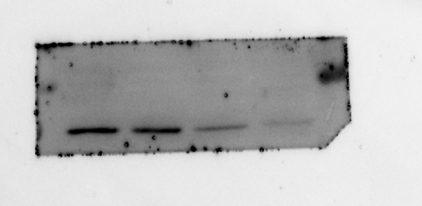

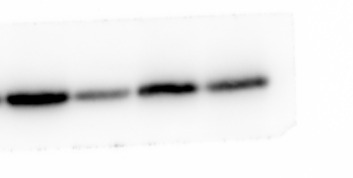

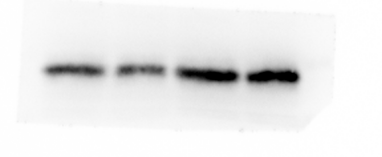

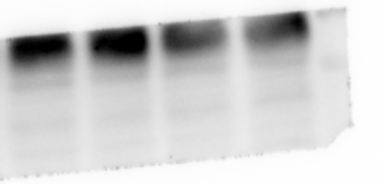

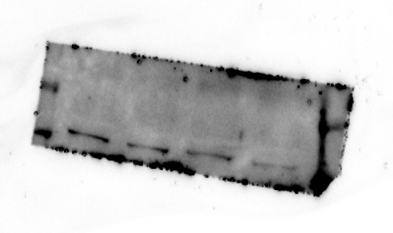

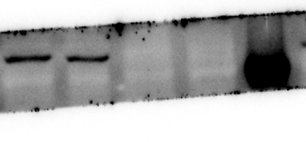

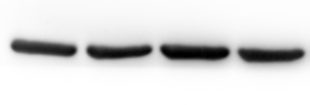


Senp1（73kD）

Nrf2（110kD）

Tfr（100kD）

Slc7a11（35kD）

Ho-1（32kD）

Ferritin（19kD）

Gpx4（20kD）

Actin（42kD）
